# Human Astrovirus in Symptomatic and Asymptomatic Children: A Cross-Sectional Study on Hospitalized and Outpatients from Rural Communities of South Africa between 2017–2021

**DOI:** 10.3390/pathogens10111398

**Published:** 2021-10-28

**Authors:** Ronewa Khumela, Jean Pierre Kabue, Afsatou Ndama Traore, Natasha Potgieter

**Affiliations:** Department of Biochemistry and Microbiology, Faculty of Science, Engineering and Agriculture, University of Venda, Private Bag X5050, Thohoyandou 0950, Limpopo Province, South Africa; ronewakhumela@gmail.com (R.K.); kabuejeanpierre@yahoo.fr (J.P.K.); afsatou.traore@univen.ac.za (A.N.T.)

**Keywords:** human astrovirus, gastroenteritis, diarrhoea, symptomatic infection, asymptomatic infection, hospitalized cases, outpatients

## Abstract

Human astroviruses are considered acute gastroenteritis agents (AGE) and are largely reported in children worldwide. There are limited data on astrovirus prevalence in rural communities, especially in hospitalized and asymptomatic cases. This study was a cross-sectional survey aiming to investigate the prevalence of classic human astroviruses in symptomatic and asymptomatic cases and hospitalized and outpatient children in rural communities of the Vhembe District, South Africa. A total of 236 stool samples (166 symptomatic and 70 asymptomatic) were collected from young children under 5 years of age. Real-time RT-PCR for astrovirus detection, RT-PCR amplification of capsid and polymerase partial genes as well as Sanger sequencing were performed. The classic astrovirus prevalence in symptomatic patients (7.23%, 12/166) as compared to healthy controls (4.29%, 3/70) was not statistically different (t-value: 1.782, *p* = 0.141: 95% CI). We did not observe a significant difference of classic astrovirus prevalence rate between the hospitalized group (6.52%, 3/46) and outpatient group (7.5%, 9/120). Symptomatic children below 6 months old were the most affected group (18.18%, 6/33). This study characterized human astrovirus genotype 2 and a putative recombinant strain (polymerase genotype 1/capsid genotype 2). Phylogenetic analysis revealed these genotypes are closely related to the strains circulating elsewhere within the African continent. The findings suggest that astrovirus is a common enteric pathogen in the study area. The results highlight the exposure of children and the need to monitor astroviruses for their potential impact in diarrhoeal diseases.

## 1. Introduction

Viral gastroenteritis, particularly diarrhoea, remains a major global health problem resulting in high morbidity and mortality especially in younger children [1]. Classic human astroviruses (HAstVs) have over the years gained global attention as important acute gastroenteritis (AGE) etiological agents, after rotavirus and norovirus [2,3]. Although they have been reported in adult AGE, astrovirus diarrhoea mainly affects the pediatric population resulting in outbreaks and sometimes hospitalization [2,4]. 

HAstVs are single-stranded, positive-sense RNA viruses which belong to the *Astroviridae* family [5]. Within the family are two genera, *Mamastrovirus* (consists of viruses infecting mammals) and *Avastrovirus* (consists of viruses infecting birds). The genome comprises three open reading frames (ORFs), ORF1a and ORF1b encoding non-structural proteins (protease and RNA-dependent RNA polymerase (RdRp)), and ORF2 which encodes the viral capsid structural protein precursor [3]. The classic group of astroviruses consists of eight genotypes (1–8), while the novel group of astroviruses is made up of two highly divergent subgroups, namely the MLB (Melbourne) with three genotypes (MLB1-3) and the VA/HMO (Virginia/Human-Mink-ovine-like) group with genotype VA1-5 [2]. Since their discovery [6], both classic and novel strains have been identified in gastroenteritis cases. However, higher rates were observed in classic human astroviruses with type 1 as the predominant genotype [2,7]. HAstV transmission is facilitated by faecal–oral route via contaminated food, water, fomites and person to person contact. Asymptomatic carriers of astrovirus may be the major reservoir responsible for the spread of the virus in any setting [5]. Astrovirus infection is usually accompanied by a two-to-three-day watery diarrhoea with or without fever and vomiting [3]. Asymptomatic infection has been scarcely reported [8,9]. 

Recent epidemiological studies in South Africa showed substantial prevalence in urban hospitalized settings [10,11,12]; however, more data which includes both symptomatic and asymptomatic carriers in hospitalized and outpatients from rural community settings are needed to estimate the total prevalence of astrovirus circulating in the country. Previous studies showed high prevalence of enteric viruses in the Vhembe District suggesting frequent exposure of children to enteric pathogens [13,14]. Therefore, this study aimed to investigate the symptomatic and asymptomatic prevalence of classic human astrovirus strains circulating in the Vhembe District among hospitalized and outpatient children.

## 2. Results

### 2.1. Study Characteristics, Clinical Features of Children and Astrovirus Prevalence

Between January 2017 and June 2021, 236 participants were recruited. Overall, more females were enrolled (52.97%, 125/236) compared to males (47.03%, 111/236). Demographic data and other sample characteristics are shown on Table 1. Throughout the sampling period, the total astrovirus prevalence was 7.23% (12/166) in outpatients and hospitalized children tested. Children in the age group 0 to 24 months accounted for many diarrhoea cases. Astrovirus infection increased with decrease in age among the outpatients. Majority of symptomatic samples were from outpatients (72.29%, 120/166) compared to hospitalized individuals (27.71%, 46/166). 

### 2.2. Living Conditions of Study Participants and Astrovirus Positive Cases

All astrovirus positive cases (12) from the symptomatic outpatients mainly presented diarrhoea followed by vomiting, however, only 2 out of 3 hospitalized individuals had dehydrating diarrhoea. There was no notable difference observed in clinical characteristics between HAstV-positive group and HAstV-negative group. However, HAstVs positive cases were frequently detected in children with poor socioeconomic background. 

**Figure 1:**
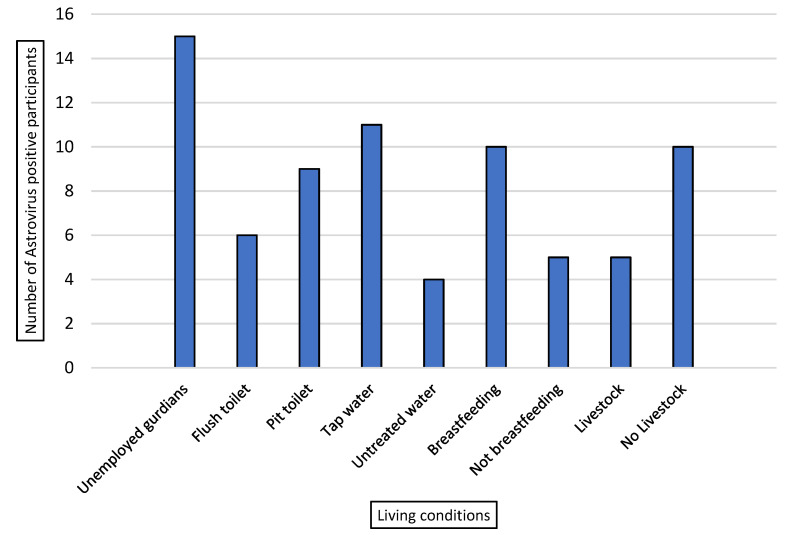
Living conditions associated with astrovirus positive cases.

All participants that tested positive for human astrovirus (15/15, 100%) were from low-income households with jobless status or unemployed guardians (Figure 1). HAstVs were detected more frequently in households using pit toilets (9/15, 60%) compared to those with flush toilets (6/15, 40%). Some individuals (4/15, 26.67%) used untreated water sources such as rivers for domestic purposes; however, the majority of participants (11/15, 73.33%) consumed municipal tap water (considered as treated water). Several astrovirus positive children (5/15, 33.33%) lived in the presence of animals/livestock. A total of 66% (10/15) of the infected children were breastfed. However, all these related living conditions were not statistically significant.

### 2.3. Comparison of Astrovirus Prevalence in Symptomatic and Asymptomatic Children

**Figure 2:**
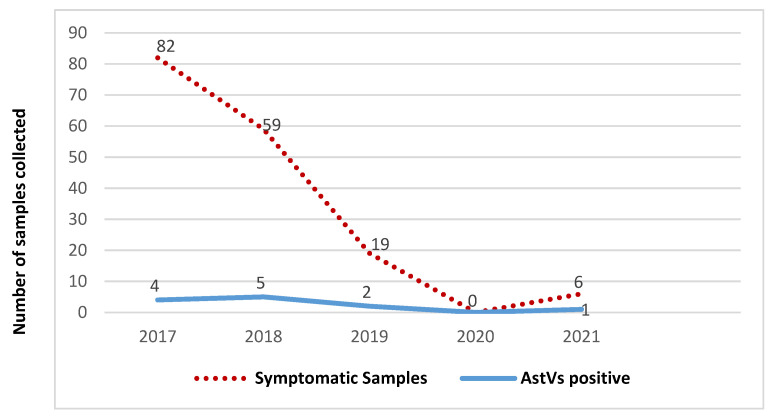
Temporal distribution of samples and astrovirus occurrence among symptomatic children.

**Figure 3:**
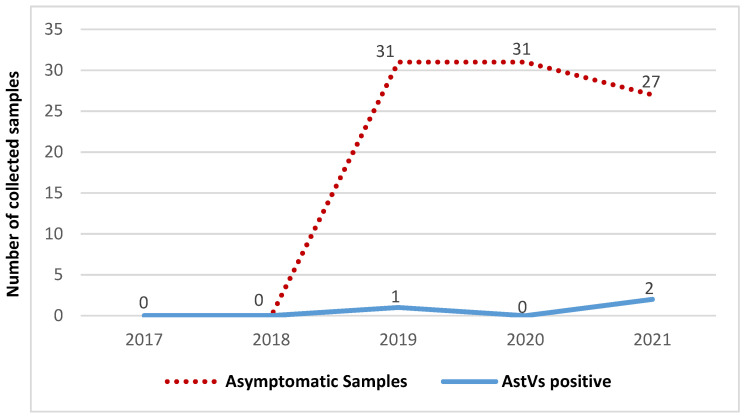
Temporal distribution of samples and astrovirus occurrence among asymptomatic children.

Out of 236 samples collected, 166 were from children presenting non-bloody diarrhoeal stools and 70 healthy controls in the Vhembe District, South Africa. Occurrence of astrovirus throughout the study period in both case and control groups is demonstrated in Figure 2 and Figure 3. No astrovirus cases were recorded in 2020 for the symptomatic group, due to limitations on sample collection during the lockdown period. There was no correlation in Ct values of positive astrovirus in both symptomatic and asymptomatic groups. Of note, the majority of positive samples had a Ct value > 30. Symptomatic patients showed higher frequency of astrovirus (7.23%, 12/166) than asymptomatic participants (4.29%, 3/70). However, there was no statistical significance in astrovirus distribution between the two groups (t-value: 1.782, *p* = 0.141: 95% CI; Table 1). 

### 2.4. Comparison of Astrovirus Prevalence in Hospitalized and Outpatient Children

From the 166 symptomatic children, 46 were hospitalized and 120 were outpatients. The difference in astrovirus prevalence in outpatients (7.5%, 9/120) and hospitalized patients (6.52%, 3/70) was not statistically significant (t-value: 1.342, *p* = 0.242: 95% CI; Table 1).

### 2.5. Genotyping and Phylogenetic Analysis

**Figure 4:**
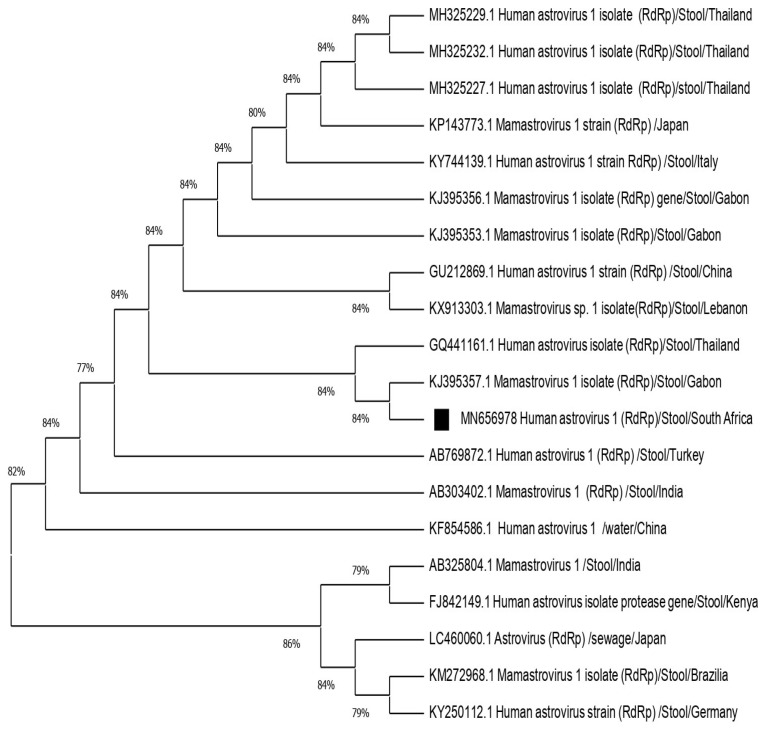
Phylogenetic tree based on 409-nucleotide sequence of HAstVs 1 polymerase gene fragment. The Neighbor-Joining test was used to set the tree. Squared black dot indicates HAstV1 genotyped in this study. The analysis involved 20 nucleotide sequences randomly selected from GenBank with their respective accession numbers. All positions containing gaps and missing data were eliminated. The evolutionary distances were computed using the *p*-distance method and are in the units of the number of base differences per site. Evolutionary analyses were conducted in MEGA X (10.0.5) and bootstrap tests (1000 replicates) based on the Kimura two-parameter model.

**Figure 5:**
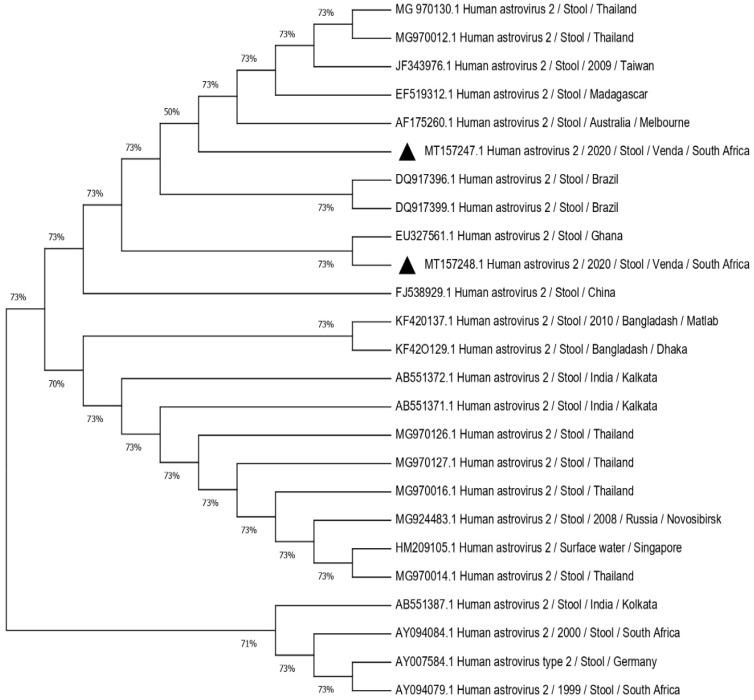
Phylogenetic tree based on the 449-nucleotide sequence of HAstVs 2 capsid gene fragment. The Neighbor-Joining tree test was used to set the tree. Black triangles indicate two HAstV type 2 genotyped in this study. A total of 23 nucleotide sequences randomly selected from GenBank with their respective accession numbers were used to set the tree. All positions containing gaps and missing data were eliminated. The evolutionary distances were computed using the *p*-distance method and are in the units of the number of base differences per site. Evolutionary analyses were conducted in MEGA X (10.0.5) and bootstrap tests (1000 replicates) based on the Kimura two-parameter model.

Out of six amplified RNA extracts (six capsid and one RdRp), two were successfully sequenced on polymerase gene fragment and capsid gene fragment generating three sequences (one with capsid genotype 2 sequence and the other one with both polymerase genotype 1/capsid genotype 2). Of note, the successfully sequenced samples were from symptomatic cases. Failure to obtain amplicons or quality sequence may be due to low viral load or RNA degradation in the samples. Most of the positive samples (12/15, 80%) showed high Ct values ranging between 31.69 to 38.39. The remaining positive samples (3/15, 20%) had Ct values < 30. The FASTA format of the sequences obtained were blasted on the GenBank repository to obtain the best hits and identify the primary sequences. The similarity with the selected reference strains ranged between 80–97%. The sequence of the RdRp gene fragment revealed the genotype of classic human astrovirus type 1 (HAstV1). The GenBank accession number for this sequence is MN656978. The remaining two partial sequences were determined to be classic human astrovirus 2 (HAstV2) in the remaining two sequences. The accession numbers for these sequences are MT157247 and MT157248. The phylogenetic tree was constructed to evaluate the genetic relationship of the identified human astrovirus strains with other related strains based on the partial nucleotide sequence of the RdRp gene fragment (Figure 4) and partial nucleotide sequence of the capsid fragment (Figure 5). The similarity amongst selected strains ranged from 80–97%. Identified strains were closely related to others circulating in the African continent.

## 3. Discussion

Gastroenteritis continues to be a global health problem with many aetiologies. The burden of human astrovirus in AGE is overlooked. The main objective of this study was to investigate the prevalence of classic human astrovirus in symptomatic and asymptomatic, outpatients and hospitalized children from rural communities of Vhembe, South Africa. In this study, we calculated total prevalence of astrovirus as we worked with both outpatients and hospitalized children. The total prevalence was 7.23% (12/166) in the Vhembe District including 7.5% (9/120) from outpatients and 6.52% (3/46) from hospitalized children. Generally, astrovirus prevalence ranges between 2% and 10% with a lower detection rate of novel astrovirus strains [2]. Different studies reporting astrovirus prevalence globally in hospitalized and outpatients demonstrated fluctuating ranges [10,11,12,15,16] with the highest recorded in Nigeria [17]. 

This investigation revealed an increased rate of HAstVs in symptomatic (7.5%) compared to asymptomatic (4.29%) patients, although the difference was not significant (t-value: 1.782, *p* = 0.141: 95% CI). Despite this statistical insignificance, the enhanced astrovirus prevalence in the symptomatic group suggests possible contribution to diarrhoeal disease in young children. Higher frequency of astrovirus prevalence in symptomatic vs. asymptomatic was reported previously [9,18]. However, Barbosa et al. (2020) recently presented equal rates between symptomatic and control group. The presence of astrovirus in asymptomatic children (4.29%) in this study reveals considerable carriage which may promote its transmission in the communities.

Although comparison between symptomatic hospitalized and outpatient children in this study did not show significant difference between both settings, the comparable prevalence rate in both settings is in favour of possible astrovirus association with hospitalization of children in rural communities. Continued surveillance in the study area over time will help to ascertain the role of astrovirus in severe AGE. Nonetheless, dehydrating diarrhoea cases were reported in only 2 out of 46 hospitalized patients. 

The majority of positive astrovirus specimens in this study were obtained from outpatients without dehydration. This may be because human astrovirus infection is commonly associated with moderate diarrhoea [19,20]. There was no association between diarrhoea cases in HAstVs positive and HAstVs negative samples. The results support the possible involvement of astrovirus in moderate cases of AGE among young children. Contrary to our findings, the MAL-ED research group reported the association of astrovirus with severe diarrhoea in a longitudinal study from several low-income countries [18].

Co-infection of enteric viruses causing AGE has been reported [20]. Multiple aetiological agents may contribute to the outcome of diarrhoeal disease [21]. Although this approach was not the scope of this study, we recommend further investigations in the involvement of astrovirus co-infection with other viral agents in AGE.

During the present investigation, children who were highly exposed to the virus were the youngest group of 0–6 months old. This trend was clearly observed in outpatients (Table 1). Similarly, Naficy et al. [22] in Egypt reported higher prevalence in age group 0–5 and 6–11 months. While astrovirus antibodies may prevent the associated symptoms, they may not necessarily stop the viral replication [23,24]. Low detection in older children may be due to protective immunity. If children particularly in this area are exposed at a younger age, they may have developed protective immunity and thus, lower infection rate could be observed in older children. Previous serological studies reported children with maternally acquired antibodies to human astrovirus early in life, by 4–6 months of age. The current findings suggest that the astrovirus vaccine development should be targeted on children in a much earlier stage of life. Further investigations should be carried out to confirm the astrovirus replication in the context of early maternal antibodies.

No correlation was observed between other symptoms associated with diarrhoea and astrovirus occurrence; however, additional factors classified under living conditions may play a role in HAstV prevalence. All child guardians were unemployed living in rural areas and thus had poor socioeconomic status with the majority using pit toilets (Figure 1). Poor society has been associated with greater levels of illness compared to well-developed society [25]. An outside water source was associated with higher cases of HAstVs in a South African study [11]. Dongdem et al. [26] detected human astrovirus in tap water. Although water quality was not tested in the current study, drinking of contaminated water and lack of hygiene practice may be associated with HAstVs occurrence [26]. 

The present study revealed the circulation of HAstV type 1 and 2. Classic human astrovirus type 1 is known as the predominant type worldwide [3,27,28]. The phylogenetic tree (Figure 4) revealed that HAstV genotype 1 from this study is closely related to strains circulating in other African countries including Gabon and Kenya [29,30]. Population movement may facilitate the importation of the strain within the African continent. 

The prevalence of type 2 HAstV is variable in different countries [31,32,33] and has reached epidemiological importance in some settings [34,35]. The phylogenetic tree (Figure 5) revealed that human astrovirus type 2 strains obtained in this study were not closely related to the previously described type 2 strains in South Africa. This indicates continuous evolution of astrovirus strains possibly due to mutation and/or recombination. Similarly, Nadan et al. (2019) observed no similarities in genotypes characterized in 2010, 2011 and 2014. The HAstV2 strain from this study is, however, related to those circulating in Ghana [36] and Asian countries [37], implying the role of population movement in the distribution of related strains.

Epidemiological studies have shown the presence of novel astrovirus in AGE worldwide [28,38,39]. However, Vu and co-workers [40], using real-time RT-PCR specific assay designed for both detection of classic and novel astroviruses, demonstrated no statistical significance when correlating the viral load with occurrence of mono or co-infection of astrovirus. 

A combined analysis of ORF1b and ORF2 regions that allow to differentiate the polymerase genotype, capsid genotype and polymerase/capsid genotype was performed in this study. Of note, a putative recombinant HAstV polymerase genotype 1/HAstV capsid genotype 2 was identified, suggesting the possible occurrence of a recombination event. More details could be obtained on the recombination event if SimPlot analysis was performed; unfortunately, the requirement for SimPlot is a long sequence of more than 1000 bp, which is not the case for our amplified strains. There is increasing evidence of recombination events in astrovirus which contribute to genetic variability and imply cross species transmission or zoonotic potential. Putative recombination sites within ORF1b and ORF2 have been reported previously [11,12,41]. It is common for ssRNA viruses to exchange genome fragments in highly conserved regions, increasing the prevalence of the virus and affecting its phylogenetic grouping and vaccine development [42]. The recombination of strains is an open door to mutations that may mislead future epidemiological investigations and compromise vaccine development. Thirty percent of astrovirus positive individuals lived in the presence of animals (Figure 1). Future investigations are needed to evaluate the involvement of astrovirus in zoonotic disease among the rural communities. Limitations of this study included the lack of seasonal distribution data of classic human astrovirus due to lockdown restrictions during the study period, and the absence of novel astrovirus strain data. Continued surveillance including both classic and novel astrovirus strains are recommended. 

## 4. Materials and Methods

### 4.1. Ethics

The study protocol and consent procedures were approved by the ethics committees of the Department of Health, Limpopo Province (Ref. 4/2/2) and the Research Directorate of the University of Venda (Ref. SMNS/12/MBY/07). Written, informed consents were given by the parents or child guardians before sample collection.

### 4.2. Study Population and Sample Collection

**Figure 6:**
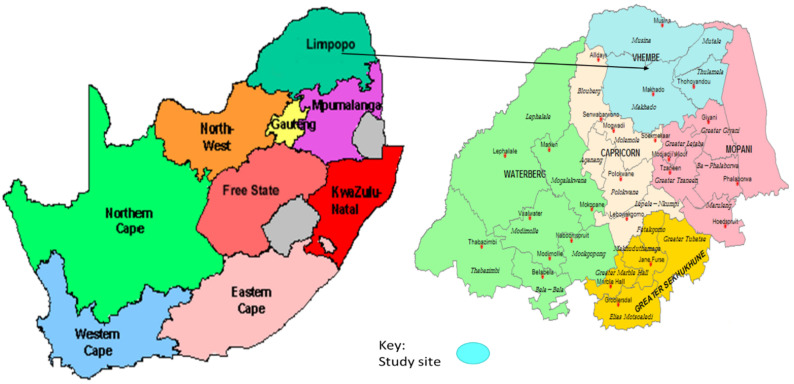
Map of South Africa (**left**) showing the Vhembe District study area (**right**) located in the Northeast region. Accessed on 24 September 2021, available on Google at: https://images.app.goo.gl/FQTF8HHiUJERfGdE7; https://images.app.goo.gl/tKi26GNUQkfL881s9.

This study was a cross-sectional investigation on children under five years of age presenting in clinics and hospitals in the Vhembe District (Figure 6). Study population was subdivided into 2 groups including: symptomatic (children with diarrhoea and other AGE symptoms such as vomiting, fever and abdominal pain) and asymptomatic (children without any AGE symptoms for at least a month). Symptomatic cases were from hospitalized children admitted within 24 h and outpatients. Diarrhoea in this study was classified as defined by the World Health Organization (WHO) as the passing of at least 3 or more loose stools within 24 h or more frequently than normal for an individual [43].

Between January 2017 and June 2021, stool samples (*n* = 236) were randomly collected from different primary health care centres within the rural communities of the Vhembe District, South Africa. All collected specimens were transported to the University of Venda Microbiology Laboratory and stored at −20 °C for further analysis. To collect data on living conditions of the participants, information such as the type of water used, the presence of livestock, type of toilet being used, breastfeeding and employment status were obtained using a questionnaire.

### 4.3. Sample Processing

#### 4.3.1. RNA Extraction

Ten percent suspension of pea sized amount of stool samples were prepared in 500 µL Phosphate Buffer Saline (PBS). RNA extraction was performed using the guanidium thiocyanate/silica method as described by Boom et al. [44]. The extracts were immediately stored at −20 °C.

#### 4.3.2. Real-Time PCR for Astrovirus Detection

Classic human astrovirus was detected using RIDA^®^GENE Viral Stool Panel II (PG1325) multiplex real-time RT-PCR assay. The test was carried out in one-step real-time RT-PCR format in which the reverse transcription of RNA is followed by the PCR in the same tube. A positive and negative control was included for each run. The kit has a detection limit of ≥50 copies per reaction and contains an Internal Control RNA (ICR) of sample preparation procedure to monitor RNA extraction efficiency and to determine possible PCR-inhibition. The real-time PCR program was performed on a Corbett Research Rotor Gene 6000 (Corbett Life Science, Sydney, Australia) with the following cycling conditions: Reverse transcription for 10 min at 58 °C; initial denaturation step for 1 min at 95 °C followed by 45 cycles of 95 °C for 15 s and 55 °C for 30 s continuous fluorescence reading. To minimize the risk of amplicons carry-over and contamination of samples, pre- and post-amplification steps were carried out in separate rooms. Samples with up to 40 of the Ct value were considered positive.

#### 4.3.3. Astrovirus RT-PCR Amplification

To confirm our astrovirus detection, a total of 6 RNA extracts that tested positive by one-step real-time RT-PCR were randomly selected for amplification using OneStep Ahead RT-PCR (QIAGEN). The primer sequences used for RT-PCR amplification were as previously described by Noel et al. (1995) and Finkbeiner et al. (2009) (Table 2). Primer specificity was checked using NCBI nucleotide blast and primer sequences gave no self-complementarity or hairpins. Primer MON269 and MON270 amplify a capsid fragment of size 449 bp in classic strains, and primers targeting the polymerase region SF0073 and SF0076 amplify a size 409 bp of both classic and novel strains of astrovirus. During OneStep Ahead RT-PCR amplification, the following optimized conditions for both sets of primers were used: Hold 1: 42 °C for 30 min; Hold 2: 95 °C for 15 min; Cycles: Denaturation—95 °C for 1 min; Annealing: 56 °C for 1 min; Extension: 72 °C for 1 min and final Extension at 72 °C for 10 min. The concentration of all primers was at 1 µM in a final reaction volume of 25 µL. 

#### 4.3.4. Genotyping and Phylogenetic Analysis of Astrovirus

The RT-PCR products of the amplified fragments were purified with Zymoclean™ Gel DNA recovery kit following manufacturer’s instructions. Using the same specific primers, Sanger sequencing was performed on ABI 3500XL Genetic Analyzer POP7™ (Thermo Fisher Scientific, Waltham, MA, USA). Raw sequence reads were edited with Finch TV v1.4 (Geospiza Inc., Seattle, WA, USA). Nucleotide sequences of HAstVs obtained were compared with reference strains obtained in the NCBI GenBank using BLAST tool available at https://www.ncbi.nlm.nih.gov/blast / {accessed on 9 March 2020} followed by construction of phylogenetic tree using MEGA X software [47]. Reference strains from GenBank which were randomly selected among the BLAST hits had ≥80% similarities with the query sequence of the strains identified. The Neighbor-Joining method [48] was used to build the phylogenetic tree and the reliability of different phylogenetic groupings was evaluated by bootstrap analysis (1000 replicates) [49]. Evolutionary distance was computed using the *p*-distance method [50].

#### 4.3.5. Data Analysis

Data were captured in Microsoft Excel 2016. A Student’s t-test was used to determine the statistical significance at *p* ≤ 0.05 between different groups.

## 5. Conclusions

The importance of astroviruses as human pathogens has increased with the widespread use of molecular techniques. With the use of real-time RT-PCR, this study confirms the occurrence of human astrovirus in young children of the Vhembe District, South Africa. The study results suggest that HAstV is a common pathogen circulating in the rural community of the Vhembe District. The astrovirus presence in this study is not predictive of diarrhoeal disease, however the findings are indicative of the exposure to astrovirus in rural settings. The prevalence of HAstV shows that this virus may be, along with other enteric pathogens, contributing to AGE in rural communities. The strains circulating in this area are closely related to other astrovirus strains which were previously reported worldwide in AGE. These findings highlight the need to consider HAstVs as important pathogens when developing preventive strategies against diarrhoea.

## Figures and Tables

**Table 1:** Sample characteristics and astrovirus prevalence.

	Symptomatic Patients						Asymptomatic Patients	
	Total Symptomatic Patients Enrolled		Outpatients		Hospitalized			
	*n*	No. of Positive (%)	*n*	Astrovirus Prevalence (%)	*n*	Astrovirus Prevalence (%)	*n*	Astrovirus Prevalence (%)
**Detection rate**	166	12 (7.23)	120	9 (7.5)	46	3 (6.52)	70	3 (4.29)
**Gender**								
Males	79	7 (8.86)	51	5 (9.80)	28	2 7.14)	32	2 (6.25)
Females	87	5 (5.74)	69	4 (5.80)	18	1 (5.56)	38	1 (2.63)
**Age groups (months)**								
0–6	33	6 (18.18)	27	5 (18.51)	6	1 (16.67)	19	1 (5.26)
7–12	52	2 (3.84)	30	1 (3.33)	22	1 (5.55)	11	0
13–24	50	2(4)	36	1 (0.83)	14	1 (7.14)	26	1 (3.85)
≥25	31	2 (6.45)	27	2 (2.78)	4	0	14	1 (7.14)

**Table 2:** Primer sequences.

Species	Code	Primers Sequence	References
HAstVs (Classic)	MON 269	F-(CAACTCAGGAAACAGGGTGT)	[45]
HAstVs (Classic)	MON 270	R-(TCAGATGCATTGTCATTGGT)	[45]
HAstVs (Classic/Novel)	SF0073	F-(ATTGGACTCGATTTGATGG)	[46]
HAstVs (Classic/Novel)	SF0076	R-(CTGGCTTAACCCACATTCC)	[46]

## Data Availability

The representative gene sequences of astrovirus obtained in the current study were submitted to GenBank under accession numbers MN656978, MT157247 and MT157248.
